# Targeting ER Stress with Saikosaponin A to Overcome Resistance under Radiation in Gastric Cancer Cells

**DOI:** 10.3390/ijms24065661

**Published:** 2023-03-16

**Authors:** Tae Woo Kim

**Affiliations:** Department of Biopharmaceutical Engineering, Dongguk University-WISE, Gyeongju 38066, Gyeongbuk, Republic of Korea; tae1410@naver.com

**Keywords:** gastric cancer, saikosaponin A, radiation, ER stress, reactive oxygen species

## Abstract

Saikosaponin A is a triterpene saponin and a potentially bioactive compound derived from *Bupleurum falcatum* L. However, the molecular mechanisms and effects of saikosaponin A in gastric cancer remain unknown. In the present study, I evaluated the effects of saikosaponin A on cell death and endoplasmic reticulum stress via calcium and reactive oxygen species release. Targeting reactive oxygen species with diphenyleneiodonium and N-acetylcysteine inhibited cell death and protein kinase RNA-like ER kinase signaling pathway by down-regulating Nox4 and inducing glucose-regulated protein 78 exosomes. Furthermore, saikosaponin A caused a synergistic inhibitory effect of the epithelial mesenchymal transition phenomenon, indicating the reversible phenotype modulation by epithelial cells under radiation exposure in radiation-resistant gastric cancer cells. These results suggest that saikosaponin A-mediated calcium and reactive oxygen species-induced endoplasmic reticulum stress overcome radio-resistance and induce cell death under radiation in gastric cancer cells. Therefore, saikosaponin A in combination with radiation may be a potential strategy for gastric cancer therapy.

## 1. Introduction

Each year, more than one million cases of gastric cancer (GC) are diagnosed worldwide, and GC is the fifth most common cancer type around the world [1]. Anti-cancer therapy aims still to kill or slow the growth of tumor cells with surgical therapy, and many therapeutic strategies, including chemotherapy, radiotherapy, cryotherapy, and immunotherapy, have been developed and challenged [2]. Chemotherapy and radiotherapy are the two most representative types of cancer treatment; however, chemo- and radio-resistance and the reduction in efficacy in GC remain serious obstacles [3,4]. Moreover, both chemotherapy and radiotherapy cause many side effects, such as the death of normal cells, fatigue, weight change, pain, gastrointestinal problems, and hair loss [5]. Therefore, herbal or natural medicine, specifically the use of natural or herbal compounds extracted from plants, may be a potential strategy to reduce the side effects of these therapies [6,7]. 

Natural medicines extracted from plants, food, and fruit have low toxicity and are normally safe, designating them potential candidates for enhancing the efficacy of anti-cancer activity [8]. Some traditional herbal medicines are known as potential anti-cancer drugs such as saikosaponin A (SSA), saikosaponin B (SSB), and saikosaponin D (SSD) extracted from *Bupleurum falcatum* L. Unlike other saikosaponin compounds, SSA and SSD are the major active compounds that exert anti-inflammatory, anti-viral, and anti-cancer effects [9]. In addition, SSA and SSD regulate cell cycle arrest, anti-proliferation, and apoptotic cell death in various cancer cell types, including colon, liver, breast, pancreatic, and lung [10,11,12,13,14]. SSA exerts antioxidant and anti-tumor effects through the induction of apoptotic cell death in liver and breast cancer [15]. SSA in combination with cisplatin treatment induces apoptotic cell death by inducing reactive oxygen species (ROS) release and inhibits the proliferation and tumorigenesis [16]. These findings suggest that SSA is a potential anti-cancer drug. 

The endoplasmic reticulum (ER) is a vital intracellular organelle in the secretory pathway and exerts many functions, including homeostasis, protein synthesis, calcium (Ca^2+^) storage, transport, degradation, folding, modification, trafficking, and lipid metabolism [17]. ER stress triggers the unfolded protein response (UPR) and is classified by three ER stress sensors, including protein kinase RNA (PKR)-like ER kinase (PERK), inositol-requiring protein-1α (IRE1α), and activating transcription factor 6 (ATF6) [18]. Under physiological stress, PERK is phosphorylated, and this signaling is prolonged by the phosphorylation of eukaryotic translation initiation factor 2 (eIF2α). The phosphorylation of eIF2α induces the activation of activating transcriptional factor 4 (ATF4) and transcriptional factor C/EBP homologous protein (CHOP) [19]. Recent reports suggest that the PERK-eIF2α-ATF4-CHOP axis overcomes radiation resistance and induces cell death in cancer cells [20]. 

Therefore, the present study aimed to evaluate the efficacy of SSA treatment in overcoming radio-resistance via the PERK–ATF4–CHOP pathway and the induction of apoptotic cell death under radiation exposure in GC cells.

## 2. Results

### 2.1. SSA Treatment Induces Apoptosis in GC Cells 

**Figure 1:**
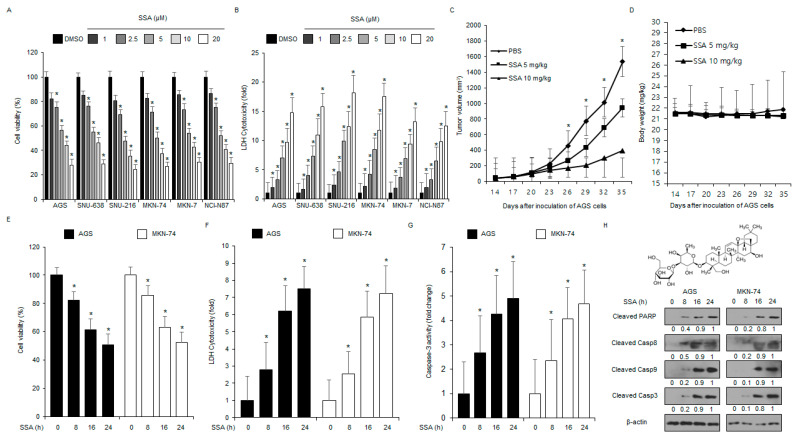
Anti-gastric cancer effect of saikosaponin A (SSA) in vitro and in vivo. (**A**,**B**) Cell viability and LDH cytotoxicity of SSA in GC cells (AGS, SNU-638, SNU-216, MKN-74, MKN-7, and NCI-N87) determined using WST-1 and LDH assays, and SSA was treated in a dose-dependent manner (0, 1, 2.5, 5, 10, and 20 µM, 24 h). Cell viability of the DMSO-treated cells was set at 100%; *, *p* < 0.05. (**C**,**D**) AGS cells (1 × 10^7^) were implanted (sc) into the thigh on the right hind leg of nude mice (*n* = 10/group). SSA (5 or 10 mg/kg) or PBS was administered (ip) once a day for two days. Body weights of the AGS tumor-xenograft mice were determined twice a week during the experiment. (**E**–**H**) The cell viability, cell cytotoxicity, and caspase-3 activity in AGS and MKN-74 cells with SSA (10 µM) in a time-dependent manner (8, 16, and 24 h) were determined using WST-1, LDH, and caspase-3 activity assays; *, *p* < 0.05. Western blotting of PARP, caspase-3, caspase-8, and caspase-9 cleavage were performed for the indicated times in SSA-treated AGS and MKN-74 cells. (**H**) The chemical structure of SSA.

To determine the cytotoxic effect of SSA on various GC cells, I performed a cell viability assay. As shown in Figure 1A,B, SSA decreased the cell viability in AGS, SNU-638, SNU-216, MKN-74, MKN-7, and NCI-N87 cells in a dose- and time-dependent manner (0, 1, 2.5, 5, 10, and 20 μM, 24 h; 0, 8, 16, and 24 h, 10 μM). To further evaluate the effects of SSA in vivo, a GC xenograft mouse model was constructed with AGS cells. Mice in the 5 mg/kg and 10 mg/kg SSA groups showed lower tumor volumes than the control group and exhibited dose-dependent efficacy (Figure 1C). Body weight did not significantly differ among the groups (Figure 1D). 

**Figure 2:**
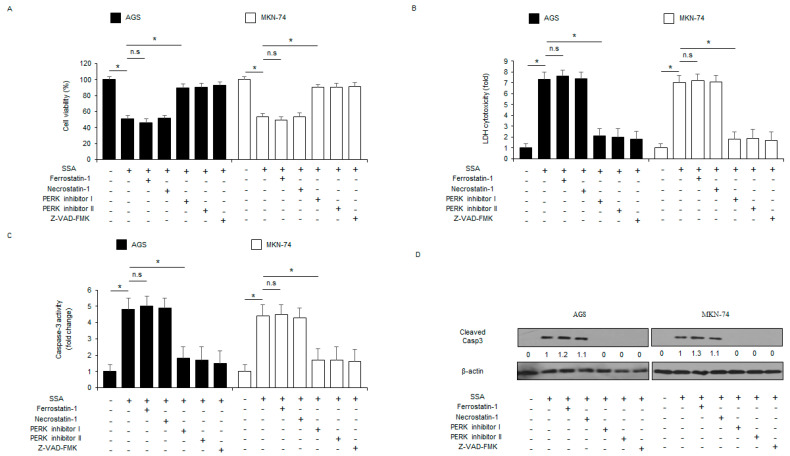
SSA regulates apoptosis and ER stress in GC cells. (**A**–**D**) The effect of a necroptosis inhibitor (necrostatin-1), a ferroptosis inhibitor (ferrostatin-1), ER stress inhibitors (PERK inhibitors I and II), and a pan-caspase inhibitor Z-VAD-FMK (50 mM, 24 h) on SSA-induced apoptotic cell death. AGS and MKN-74 cells were pretreated with ferrostatin-1 (2 μM, 24 h), necrostatin-1 (20 μM, 24 h), PERK inhibitor I (10 μM, 24 h), PERK inhibitor II (10 μM, 24 h), and Z-VAD-FMK (50 mM, four hours) and then treated with SSA (10 µM, 24 h). WST-1, LDH, and caspase-3 activity assays were performed; *, *p* < 0.05. Total lysates were subjected to Western blot assay to identify apoptosis marker caspase-3 cleavage. β-actin was used as a protein loading control.

To evaluate the cytotoxic effect of SSA, lactate dehydrogenase (LDH) and caspase-3 activity assays were conducted at the indicated times (0, 8, 16, and 24 h). As shown in Figure 1E–G, LDH release and caspase-3 activity were time-dependently increased in SSA (10 μM; 0, 8, 16, and 24 h)-treated AGS and MKN-74 cells. Cell viability was also time-dependently inhibited in these cells. I also investigated whether SSA modulated caspase-dependent cell death using Western blot analyses. SSA treatment enhanced pro-apoptotic proteins, including PARP, caspase-3, caspase-8, and caspase-9 cleavage at various time points (Figure 1H). To verify whether SSA-mediated cell death could be blocked by various cell death inhibitors, I treated AGS and MKN-74 cells with SSA (10 μM, 24 h), a ferroptosis inhibitor (ferrostatin-1; 2 μM, 24 h), a necroptosis inhibitor (necrostatin-1; 20 μM, 24 h), PERK inhibitor I (10 μM, 24 h), PERK inhibitor II (10 μM, 24 h), and a pan-caspase inhibitor (Z-VAD-FMK; 50 µM, 24 h). My findings showed that PERK inhibitor I, PERK inhibitor II, and Z-VAD-FMK treatment suppressed the reduction in cell viability and the increase in LDH release and caspase-3 activity in SSA-treated GC cells (Figure 2A–C). Western blot analyses indicated that SSA in combination with PERK inhibitor I, PERK inhibitor II, or Z-VAD-FMK, reduced SSA-mediated cleaved caspase-3 levels (Figure 2D).

### 2.2. SSA Mediates Apoptotic Cell Death through the ER Stress Pathway in GC Cells

**Figure 3:**
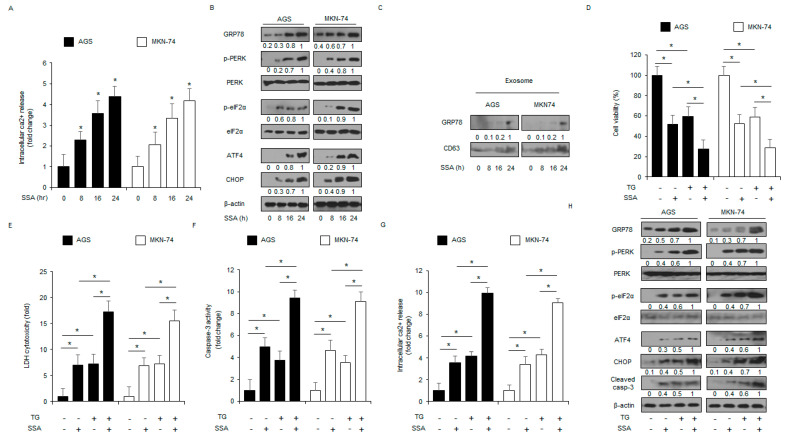
SSA induces ER stress in GC cells. (**A**,**B**) AGS and MKN-74 cells were treated with SSA (10 μM) for the indicated times and an intracellular Ca^2+^ assay was performed; *, *p* < 0.05. ER stress markers (GRP78, p-PERK, PERK, p-eIF2α, eIF2α, ATF4, and CHOP) were measured by Western blot assay. Β-actin was used as a protein loading control. (**C**) AGS and MKN-74 cells were treated with SSA (10 μM) for the indicated times, and then exosomes (30 μg) were collected from the cell supernatant. Total exosomes were determined by Western blotting using the exosome marker, CD63, and the ER stress marker, GRP78. (**D**–**H**) Cell viability, LDH release, caspase-3 activity, intracellular Ca^2+^, and GRP78, p-PERK, PERK, p-eIF2α, eIF2α, ATF4, CHOP, and cleaved caspase-3 levels were determined in thapsigargin (TG; 3 μM, 24 h) and SSA (10 μM, 24 h)-treated AGS and MKN-74 cells; *, *p* < 0.05.

Emerging studies indicate that ER stress leads to cell death and overcomes radio-resistance in cancer [21]. To confirm whether SSA induces ER stress signaling in GC cells, I measured SSA-triggered Ca^2+^ release. When intracellular Ca^2+^ release assay was performed, SSA induced intracellular Ca^2+^ release in a concentration- and time-dependent manner (Figure 3A). To affirm the levels of SSA-treated ER stress markers, such as GRP78, p-PERK, PERK, p-eIF2α, eIF2α, ATF4, and CHOP in a time-dependent manner, Western blot analyses were performed. SSA treatment induced the upregulation of GRP78, p-PERK, p-eIF2α, ATF4, and CHOP levels (Figure 3B). Emerging reports suggest that GRP78 is located in the exosome fractions in cancer cells and is up-regulated during the ER stress response [22]. To study the function of GRP78 on SSA-mediated exosome release, I treated AGS and MKN-74 cells with SSA and extracted the secreted exosomes from their culture supernatants. SSA increased the levels of the exosome marker CD63 in a time-dependent manner, and GRP78 was notably upregulated in exosomes extracted from SSA-treated cell culture medium compared to that from the control (Figure 3C). Our results suggest that GRP78 + exosomes are an important for SSA-triggered ER stress and cell death. I also assessed the effect of SSA in combination with the ER stress inducer thapsigargin (TG) on GC cells. Combination experiments showed that both TG and SSA reduced cell viability and enhanced LDH release, caspase-3 activity, and intracellular Ca^2+^ release compared with the control (Figure 3D–G). In addition, SSA, in combination with TG, increased GRP78, p-PERK, p-eIF2α, ATF4, CHOP, and cleaved caspase-3 levels (Figure 3H). 

### 2.3. The Inhibition of ER Stress Signaling Blocks SSA-Induced Apoptotic Cell Death 

**Figure 4:**
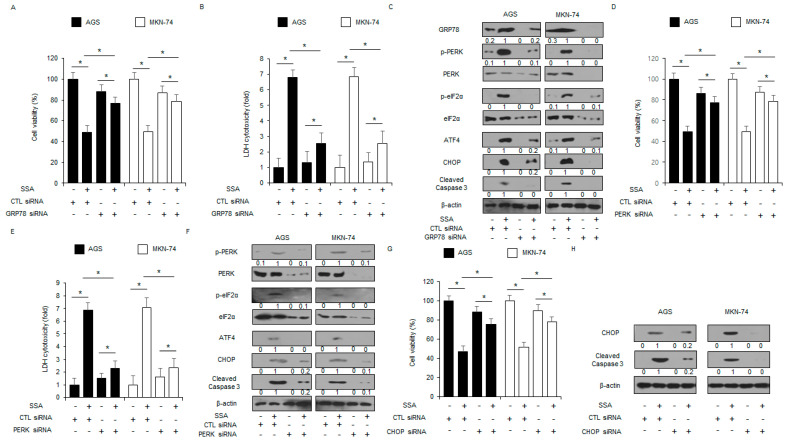
Targeting ER stress inhibits SSA-induced apoptotic cell death in GC cells. (**A**–**C**) AGS and MKN-74 cells were transfected with GRP78 siRNA with SSA treatment (10 μM, 24 h). Then, cell viability, LDH release, and GRP78, p-PERK, PERK, p-eIF2α, eIF2α, ATF4, CHOP, and cleaved caspase-3 levels were determined; *, *p* < 0.05. β-actin was used as a protein loading control. (**D**–**F**) Cell viability, LDH release, and p-PERK, PERK, p-eIF2α, eIF2α, ATF4, CHOP, and cleaved caspase-3 levels in AGS and MKN-74 cells treated with SSA (10 μM, 24 h) were determined in the presence or absence of PERK siRNA (30 nM, 24 h); *, *p* < 0.05. β-actin was used as a protein loading control. (**G**,**H**) Cell viability and Western blot analyses for CHOP and cleaved caspase-3 in AGS and MKN-74 cells treated with SSA (10 μM, 24 h) were performed in the presence or absence of CHOP siRNA (30 nM, 24 h); *, *p* < 0.05. β-actin was used as a protein loading control.

To confirm whether GRP78 from the cell lysate and exosomes regulates SSA-mediated apoptotic cell death in GC cells, a GRP78 knockdown experiment was performed. GRP78 knockdown triggered the increase in cell viability and the reduction in LDH release in SSA-treated GC cells compared to control cells (Figure 4A,B). Compared to controls with SSA treatment, GRP78 knockdown with SSA treatment inhibited GRP78, p-PERK, p-eIF2α, ATF4, CHOP, and cleaved caspase-3 (Figure 4C). The PERK-CHOP axis is a potential factor for cell death in cancer and chemoresistance [23]. I carried out a PERK knockdown study using a PERK-specific siRNA in SSA-treated GC cells. These cells were transfected with PERK siRNA (30 nM, 24 h) and treated with SSA. In the control cells, SSA decreased cell viability and enhanced LDH release, whereas SSA-treated PERK knockdown cells showed higher cell viability and lower LDH release than control cells (Figure 4D,E). Western blot analyses indicated that control cells had increased p-PERK, p-eIF2α, ATF4, CHOP, and cleaved caspase-3 with SSA treatment, whereas PERK-knockdown cells showed decreased p-PERK, p-eIF2α, ATF4, CHOP, and cleaved caspase-3 with SSA treatment (Figure 4F). In a CHOP- knockdown experiment, SSA treatment reduced cell viability and increased LDH release in control cells, whereas SSA-treated CHOP knockdown cells showed higher cell viability and lower LDH release than control cells (Figure 4G). Western blot analyses showed that control cells had increased levels of CHOP and cleaved caspase-3 with SSA treatment, whereas PERK-knockdown cells had decreased levels of CHOP and cleaved caspase-3 with SSA treatment (Figure 4H). 

### 2.4. SSA Induces ER Stress and Cell Death via ROS and Ca^2+^ Generation in GC Cells

**Figure 5:**
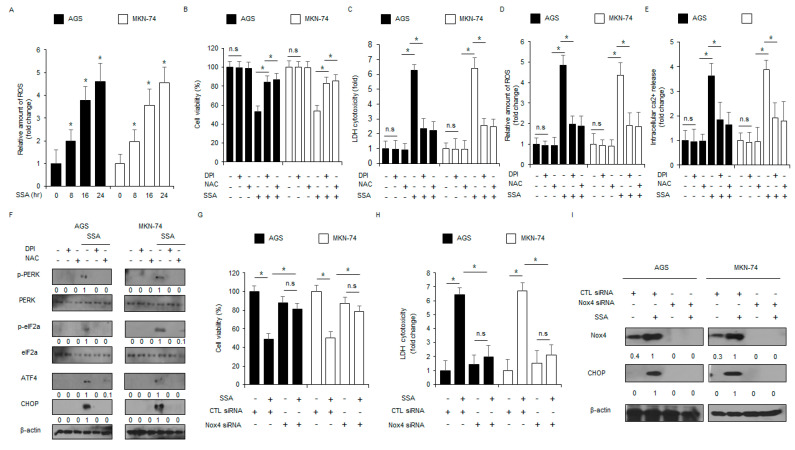
Nox4 regulates ER stress and cell death in SSA-treated GC cells. (**A**) AGS and MKN-74 cells were treated with SSA (10 μM) for the indicated times, and the cells were stained with DCFDA (20 μM) and analyzed with a microplate reader. (**B**–**F**) AGS and MKN-74 cells were pre-treated with NAC (10 mM) and DPI (10 μM) for four hours and then treated with SSA (10 μM) for 24 h. Cell viability, cell cytotoxicity, ROS release, and Ca^2+^ release were determined using WST-1, LDH, intracellular ROS, and intracellular Ca^2+^ assays; *, *p* < 0.05. ER stress markers (p-PERK, PERK, p-eIF2α, eIF2α, ATF4, and CHOP) were determined by Western blot assay. β-actin was used as a protein loading control. (**G**–**I**) After AGS and MKN-74 cells were transfected with Nox4 siRNA, they were treated with SSA (10 μM, 24 h), and cell viability, cytotoxicity, and Nox4 and CHOP levels were determined; *, *p* < 0.05. β-actin was used as a protein loading control.

Many reports suggest that the accumulation of reactive oxygen species (ROS) induces ER stress and cell death in cancer cells [24]. To identify whether SSA induces ROS production, I performed a cellular ROS assay after staining with the fluorescent probe, DCFDA. After a 16-hour treatment with SSA, DCFDA fluorescence was enhanced by 3.5–4-fold in AGS and MKN-74 cells when compared to control cells (Figure 5A). To assess whether SSA-induced ROS release was related to ER stress and cell death in AGS and MKN-74 cells, ROS inhibitors, including NAC and DPI, were co-treated with SSA. As shown in Figure 5B–F, both NAC and DPI blocked SSA’s ability to decrease cell viability and enhance LDH cytotoxicity, ROS release, and Ca^2+^ production. Western blot analyses revealed that NAC and DPI blocked the SSA-mediated increase in p-PERK, p-eIF2α, ATF4, and CHOP in AGS and MKN-74 cells. Emerging reports suggest that NADPH oxidase Nox4 is a powerful regulator for ROS release, and the Nox4-ROS pathway regulates cell survival and cell death in cancer [25]. To identify whether SSA modulates ROS and ER stress by activating Nox4, I performed knockdown experiments using Nox4-specific siRNA in AGS and MKN-74 cells, cell viability and LDH assays, and Western blot analyses. My results indicate that SSA did not reduce cell viability, and it did not enhance LDH cytotoxicity in Nox4 knockdown cells when compared to control cells (Figure 5G,H). After quantifying the Nox4 knockdown in AGS and MKN-74 cells, Western blot analyses showed that SSA treatment up-regulated Nox4 and CHOP expression in control cells. However, in Nox4 knockdown cells, SSA treatment decreased the expression of Nox4 and CHOP (Figure 5I).

### 2.5. SSA Treatment Overcomes Radio-Resistance in GC Cells

**Figure 6:**
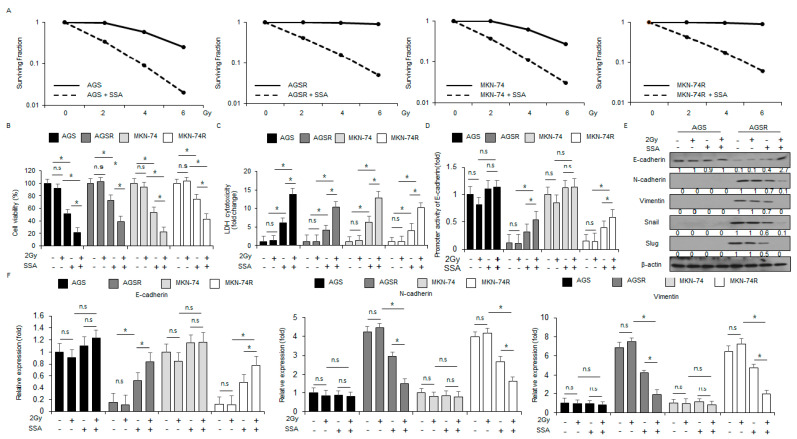
SSA, in combination with 2 Gy, inhibits the EMT phenotype in radio-resistant GC cells. (**A**) A colony assay was performed with SSA (10 μΜ, 24 h) treatment after exposure to the indicated radiation doses (0, 2, 4, or 6 Gy). The survival fraction was analyzed using the surviving fraction formula in AGS, MKN-74, AGSR, and MKN-74R cells; *, *p* < 0.05. (**B**,**C**) AGS, MKN-74, AGSR, and MKN-74R cells were treated with SSA (10 μΜ, 24 h) after 2 Gy radiation exposure. Cell viability and cell cytotoxicity were determined using WST-1 and LDH assays. (**D**) AGS, MKN-74, AGSR, and MKN-74R cells were transfected with a reporter luciferase vector containing an E-cadherin promoter (−368~+51), and were exposed to 2 Gy and SSA (10 μM, 24 hours). The luciferase activity was normalized to the activity of Renilla luciferase; *, *p* < 0.05. (**E**,**F**) Western blot analyses for E-cadherin, N-cadherin, vimentin, Slug, and Snail were performed and then E-cadherin, N-cadherin, and vimentin mRNA levels were measured using real-time RT-PCR.

To investigate whether SSA has an anti-cancer effect in radio-resistant GC cells, I performed colony formation assays, cell viability assays, and Western blot analyses. These results indicated that SSA treatment induces a synergistic reduction in the surviving fraction at the indicated radiation exposure (2, 4, or 6 Gray (Gy)) in GC (AGS and MKN-74) and radio-resistant GC cells (AGSR and MKN-74R) when compared to control cells (Figure 6A). Surviving fraction levels were lower in SSA-treated AGS and MKN-74 cells compared to SSA-treated AGSR and MKN-74R cells (Figure 6A). In AGS and MKN-74 cells, SSA reduced cell viability and increased LDH cytotoxicity, and SSA/2 Gy declined cell viability and enhanced LDH cytotoxicity even further, while 2 Gy alone had no effect (Figure 6B,C). In AGSR and MKN-74R cells, SSA treatment decreased cell viability and increased LDH cytotoxicity, and SSA/2 Gy reduced cell viability and increased LDH cytotoxicity even further, while 2 Gy alone also had no effect (Figure 6B,C). In addition, SSA or SSA/2 Gy triggered greater cell viability and lower LDH cytotoxicity in AGSR and MKN-74R cells compared to AGS and MKN-74 cells (Figure 6B,C). I next constructed a luciferase reporter driven by the E-cadherin promoter. A luciferase assay indicated increased activity of the E-cadherin promoter in 2 Gy and SSA-exposed AGSR and MKN74R cells (Figure 6D). To examine whether SSA treatment affects the EMT phenomenon in radiation-exposed GC and radio-resistant GC cells, I treated AGS, MKN-74, AGSR, and MKN-74R cells with a combination of SSA and 2 Gy radiation. Western blot analyses showed that SSA and 2Gy/SSA treatments blocked N-cadherin, vimentin, Snail, and Slug and enhanced E-cadherin in AGSR cells, whereas they remained largely unchanged in AGS cells (Figure 6E). Real-time RT-PCR indicated that 2Gy/SSA down-regulated Slug and Snail mRNA levels and up-regulated E-cadherin in AGSR and MKN-74R cells compared to control cells (Figure 6F). These findings showed that SSA induced cell death by inhibiting the EMT phenomenon in AGSR and MKN-74R cells. 

### 2.6. Targeting PERK Blocks SSA-Induced Cell Death under Radiation Exposure in GC Cells

**Figure 7:**
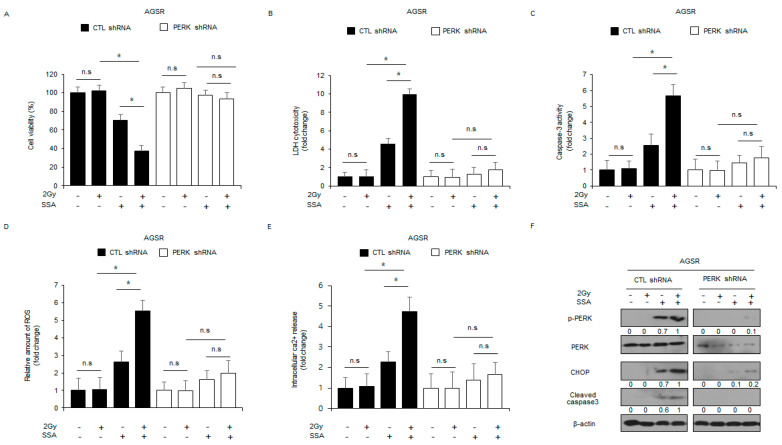
Targeting PERK inhibits apoptotic cell death in radio-resistant GC cells subjected to SSA in combination with 2 Gy. (**A**–**F**) PERK shRNA stable AGSR cell lines were established after AGSR cells were transfected with PERK shRNA. These cells were exposed to SSA (10 μΜ), 2 Gy, and SSA/2 Gy, and cell viability, cell cytotoxicity, caspase-3 activity, intracellular ROS release, and intracellular Ca^2+^ release were determined along with Western blot analyses examining the levels of p-PERK, PERK, CHOP, and cleaved caspase-3; *, *p* < 0.05. β-actin was used as a protein loading control.

To further determine whether PERK signaling regulates SSA-induced cell death under radiation exposure in radio-resistant GC cells, I established stable PERK-knockdown cells. I treated these cells with 2 Gy, SSA, and SSA/2 Gy and then performed cell viability, LDH cytotoxicity, caspase-3 activity, ROS release, Ca^2+^ release, and Western blot assays. These results indicated that SSA decreased cell viability and increased LDH release, caspase-3 activity, ROS release, and Ca^2+^ release in control cells, whereas 2 Gy had no effect. SSA/2 Gy decreased cell viability and increased LDH release, caspase-3 activity, ROS release, and Ca^2+^ release more effectively than SSA alone; conversely, in stable PERK-knockdown AGSR cells, SSA, 2 Gy, and SSA/2 Gy had no effect on cell viability, LDH cytotoxicity, caspase-3 activity, ROS release, and Ca^2+^ release (Figure 7A–E). In control cells, Western blot analyses showed that SSA treatment up-regulated the expression of p-PERK, CHOP, and cleaved caspase-3, and SSA/2 Gy up-regulated the expression of p-PERK, CHOP, and cleaved caspase-3 (Figure 7F). In PERK-knockdown AGSR cells, SSA and SSA/2 Gy did not affect the expression of p-PERK, CHOP, and cleaved caspase-3 (Figure 7F). Together, our findings suggest that PERK activation by SSA regulates ER stress-mediated cell death under radiation exposure in GC cells and radio-resistant GC cells. 

## 3. Discussion

In the present study, I identified apoptotic cell death mechanism via ROS and ER stress in SSA-treated GC cells. In addition, SSA/2 Gy exerted synergistic anti-tumor effects by activating ER stress and caspase-dependent cell death and by inhibiting the EMT phenomenon in radio-resistant GC cells. 

ER stress via the UPR signaling pathway mediates cellular protein homeostasis and induces cell survival of damaged and stressed cells [26]. However, prolonged and excessive ER stress induces cellular dysfunction and apoptotic cell death by releasing ROS. Overwhelming the UPR and ER stress disturbs the general roles of the ER, such as homeostasis [27,28,29]. The main UPR-PERK signaling pathway (PERK–eIF2α–ATF4–CHOP axis) phosphorylates eIF2α under ER stress [30]. The phosphorylation of eIF2α induces translation and the nucleus translocation of ATF4, which then mediates apoptotic cell death via the activation of CHOP [31]. Recent studies reported that excessive ER stress induces caspase-dependent apoptotic cell death via Ca^2+^ release from the ER [32]. Increased Ca^2+^ production causes the accumulation of unfolded proteins that trigger ER stress by disturbing ER function [33]. Apoptotic cell death by Ca^2+^ release regulates Bcl-2, and the suppression of Bcl-2 induces apoptotic cell death by regulating the Ca^2+^-dependent serine-threonine phosphatase calcineurin [34]. The interaction between the Ca^2+^-binding chaperone, calcineurin, and the UPR sensor, PERK, is increased by releasing Ca^2+^, calcineurin knockdown inhibits ER stress-induced cell death [35]. 

Bioactive compound saikosponins, such as SSA, SSB, SSC, and SSD extracted from Radix Bupleuri, have various in vitro and in vivo effects, including anti-cancer, anti-inflammatory, and anti-viral functions [36]. Many studies reported that SSD exerts powerful anti-tumor effects and induces apoptotic cell death via diverse molecular mechanisms [37,38]. SSD induces apoptotic cell death through cell cycle arrest at G-1 phase in prostate cancer, thyroid carcinoma, non-small cell lung cancer, and renal cell carcinoma [39,40,41,42]. SSD, in combination with radiation (2 Gy), mediates powerful anti-cancer effects by inducing G-0/G-1 arrest, by upregulating p53 and Bax expression, and by downregulating Bcl2 expression under hypoxia exposure in SMMC-7721 hepatocellular carcinoma cells [43]. A Ca^2+^ agent, in combination with SSD, overcame chemoresistance via p53 overexpression and induced G2/M arrest, mitochondrial fission, and apoptotic cell death via Ca^2+^ release in ovarian cancer cells [16]. In addition, a novel sarcoplasmic/endoplasmic reticulum Ca^2+^-ATPase (SERCA) inhibitor, in combination with SSD, induced ER stress and autophagic cell death by releasing Ca^2+^ [44]. SSA mediated apoptotic cell death via cell cycle arrest at sub-G1 phase, the caspase-9/-3 axis, the upregulation of pro-apoptotic proteins, such as Bak, Bad, and PUMA, and the downregulation of Bcl-2 expression in the rat HSC cell line, HSC-T6, and the human HSC cell line, LX-2 [45]. SSA, in combination with doxorubicin, vincristine, or paclitaxel overcomes chemoresistance by downregulating P-glycoprotein (P-gp) in multidrug resistance (MDR) overexpressing MCF-7/ADR and HepG2/ADM cells [46]. SERCA is a Ca^2+^ transporter located on the sarcoplasmic reticulum (SR) and ER, and the disruption of Ca^2+^ homeostasis by SERCA inhibition induces apoptotic cell death by activating ER stress, Ca^2+^ accumulation, cytochrome C release, and caspase cleavage [47]. In the present study, I identified that SSA is a novel SERCA inhibitor along with SSD and thapsigargin, and that SSA mediates ER stress and apoptotic cell death via ROS release, Ca^2+^ release, LDH release, and the upregulation of Nox4, and caspase activity in GC cells. Furthermore, combined therapy with SSA and radiation (2 Gy) overcomes radio resistance by inhibiting the EMT phenotype; however, in PERK knockdown radioresistant AGSR cells, SSA/2 Gy inhibited apoptotic cell death by inhibiting the PERK-ATF4-CHOP axis. 

In conclusion, my findings indicate that SSA induces ER stress-mediated cell death by releasing ROS and Ca^2+^ in GC and radio-resistant GC cells. Moreover, these results suggested that SSA may be a novel anti-tumor therapeutic strategy for radiotherapy.

## 4. Materials and Methods

### 4.1. Reagents

Saikosaponin A (SSA; S8946), ferrostatin-1 (SML0583), necrostatin-1 (N9037), a PERK inhibitor (GSK2606414; 516535), 3-MA (M9281), Z-VAD-FMK (a caspase inhibitor; V116), thapsigargin (TG; ER stress inducer; T9033), diphenyleneiodonium (DPI; a Nox inhibitor; D2926), and N-acetylcysteine (NAC; a ROS inhibitor; A7250) were purchased from Sigma-Aldrich (St. Louis, MO, USA). 

### 4.2. Cell Culture 

Human GC cell lines (AGS, SNU-638, SNU-216, MKN-74, MKN-7, and NCI-N87) were purchased from the Korean Cell Line Bank (Cancer Research Center, Seoul National University, Seoul, Republic of Korea). Cells were cultured in DMEM or RPMI1640 medium (Welgene, Daegu, Republic of Korea) supplemented with 10% fetal bovine serum (FBS), 100 U/mL penicillin and 100 mg/mL streptomycin (Welgene, Republic of Korea) at 37 °C under a humidified 95%/5% (*v*/*v*) mixture of air and CO_2_.

### 4.3. Cell Viability 

Cell viability was measured using the WST-1 assay (Roche Applied Science, Indianapolis, IN, USA). The absorbance of each well was measured at 450 nm using a microplate reader (Molecular Devices, CA, USA). 

### 4.4. LDH Assay 

Cells were seeded onto a 96-well plate in growth medium, and cell cytotoxicity was analyzed with the Pierce lactate dehydrogenase (LDH) cytotoxicity assay kit according to the manufacturer’s protocol (Thermo Scientific, Waltham, MA, USA). 

### 4.5. Caspase Activity Assay 

Cells were seeded onto a 96-well plate in growth medium, and caspase-3 activity was analyzed using the caspase-3 activity assay kit (colorimetric) (Abcam, Milpitas, CA, USA) according to the manufacturer’s instructions. 

### 4.6. Ionizing Radiation (IR) 

IR exposure (2, 4, and 6 Gray (Gy)) was performed using ^137^Cs as a radiation source (Atomic Energy of Canada, Ltd., Mississaga, ON, Canada). 

### 4.7. Development of Acquired Radio-Resistant GC Cell Lines

AGS and MKN-74 cells were seeded and cultured to approximately 50~60% confluence and exposed to 2 Gy radiation daily for three months. Then, radio-resistant GC cell lines (AGSR and MKN-74R) were established from the respective parental cell lines (AGS and MKN-74). 

### 4.8. Colony Formation Assay 

GC Cells (AGS, AGSR, MKN-74, and MKN-74R) were plated and incubated onto 60 mm dishes at a density of 1000 cells/dish. After incubation, colonies were fixed and stained with 1% methylene blue in 50% ethanol. The survival fraction was calculated using the following formula: surviving fraction = number of colonies formed/number of cells seeded x plating efficiency of the control group.

### 4.9. Transfection 

GC cells in a 6-well plate were transfected with double-stranded siRNAs (30 nmol/mL, Santa Cruz) against GRP78 (Santa Cruz; sc-29338), PERK (Santa Cruz; sc-36213), Nox4 (Santa Cruz; sc-41586), and CHOP (Bioneer; 1649-1) and shRNAs against PERK (Santa Cruz; sc-36213-SH) for 24 h using Lipofectamine 2000 reagent (Invitrogen, Carlsbad, CA, USA) according to the manufacturer’s protocol. 

### 4.10. Isolation of Total RNA 

Total RNA from GC cells in a 100 mm cell culture dish was isolated using Trizol reagent according to the manufacturer’s protocols (Invitrogen, Carlsbad, CA, USA). 

### 4.11. Real-Time RT-PCR 

PCR reactions were performed in triplicate for each sample using an ABI Power SYBR green PCR Master Mix (Applied Biosystems, Foster City, CA, USA) with E-cadherin-specific primers [5′-GAACGCATTGCCACATACAC-3′ (sense) and 5′-GAATTCGGGCTTGTTGTCAT-3′ (antisense) and vimentin-specific primers (5′-CCAGGCAAAGCAGGAGTC-3′ (sense) and 5′-CGAAGGTGACGAGCCATT-3′ (antisense)] on a Roche Light Cycler 96 System (Roche, Mannheim, Germany). RNA quantity was normalized to β-actin primers [5′-AAGGCCAACCGCGAGAAGAT-3′ (sense) and 5′-TGATGACCTGGCCGTCAGG-3′ (antisense)], and gene expression was quantified according to the 2^-ΔΔCt^ method.

### 4.12. Luciferase Reporter Assay

AGS, AGSR, MKN-74, and MKN-74R cells (1 × 10^5^ cell/well) were seeded in a 6-well plate and transfected with 2 μg pGL2 luciferase vector (Promega, Madison, WI, USA) and reporter luciferase vector containing an E-cadherin promoter (−368~+51) after 24 hours using Lipofectamine 2000 agent (Invitrogen, Carlsbad, CA, USA). Luciferase activity was measured using a dual-luciferase reporter assay kit (Promega, Madison, WI, USA) on Molecular Devices Filter Max F3 (Molecular Devices, CA, USA). The luciferase activity was normalized to the activity of the Renilla luciferase.

### 4.13. Isolation of Protein

Protein cell lysates from GC cells (2 × 10^6^ cell/well) were collected in RIPA buffer containing a protease inhibitor cocktail (Sigma-Aldrich, St. Louis, MO, USA). The supernatant was analyzed for protein content using the BCA method (Thermo Scientific, CA, USA). 

### 4.14. Western Blotting Analyses

Equal amounts of protein (20 μg) were size-fractionated by 8–15% SDS-PAGE and then transferred onto a nitrocellulose membrane (Millipore Corporation, Billerica, MA, USA). The following primary antibodies (1:1000) were used: β-actin (Santa Cruz; sc-47778), eIF2α (Santa Cruz; sc-133132), Nox4 (Proteintech, 14347-1-AP), CD63 (Abcam, ab216130), cleaved PARP (CellSignaling, #5625), cleaved caspase-3 (CellSignaling, #9661), cleaved caspase-8 (CellSignaling, #9748), cleaved caspase-9 (CellSignaling, #20750), E-cadherin (CellSignaling, #3195), N-cadherin (CellSignaling, #13116), vimentin (CellSignaling, #5741), Slug (CellSignaling, #9585), Snail (CellSignaling, #9585), GRP78 (CellSignaling, #3177), PERK (CellSignaling, #5683), p-PERK (Thr980) (CellSignaling, #12185), p-eIF2α (Ser51) (CellSignaling, #3398), ATF4 (CellSignaling, #11815), and CHOP (CellSignaling, #2895). The following secondary antibodies were used: mouse anti-rabbit IgG HRP-linked antibody (Santa Cruz, 1:6000, sc-2357) and rabbit anti-mouse IgG HRP-linked antibody (Santa Cruz, 1:6000, sc-358914). The blots were visualized using the D-Plus ECL Pico System (DonginLS, Seoul, Republic of Korea, ECL-PS100). 

### 4.15. Measuring of Reactive Oxygen Species (ROS)

ROS generation was measured after staining for 45 min with the DCFDA Cellular ROS Assay Kit (Abcam, Cambridge, MA, USA; ab113851). DCF fluorescence was immediately analyzed using a microplate reader (Molecular Devices, CA, USA). 

### 4.16. Exosomes Isolation

Exosomes were obtained from the supernatant of SSA (10 μM)-treated GC cells according to the manufacturer’s protocol (Total Exosome Isolation Reagent for cell culture media, Thermo Scientific, CA, USA). 

### 4.17. Intracellular Ca^2+^ Assays

AGS and MKN-74 cells were plated into a 96-well plate with growth medium at 1 × 10^4^ cells/well. After 24 h, the cells were treated with SSA for 24 h. An intracellular Ca^2+^ activity assay [Abcam, Ca^2+^ Assay Kit (Colorimetric)] was performed and analyzed as described in the supplier’s manual (Abcam, Cambridge, MA, USA). 

### 4.18. Animals

For the animal study, five-week-old, female, athymic BALB/c nude mice (*nu/nu*) were purchased from OrientBio, Inc. (Daejeon, Republic of Korea), and maintained for 1 week with free access to sterile standard mouse chow (NIH-7 open formula) and water before use. Mice were housed randomly at 50 ± 20% humidity and approximately 21 ± 2 °C on a 12 h light–dark cycle (*n* = 10 mice/group). All animal experimental procedures were performed according to the National Institutes of Health guidelines and a protocol approved by the Institutional Animal Care and Use Committee of Kyung Hee University (KHSASP-20-250).

### 4.19. Tumor Xenograft Mouse Models

For the mice xenograft experiment, mice, aged six weeks, were inoculated with an AGS human gastric cancer cell line by subcutaneously (sc) implanting 1 × 10^7^ cultured cells into the right thigh. Six days later, mice were grouped randomly (*n* = 10 per group) and SSA (5 or 10 mg/kg) was administered intraperitoneally (ip) every other day. Tumor sizes on two axes (*L,* longest axis; *W,* shortest axis) were measured three times per week using Vernier calipers. Tumor volume was calculated as (*L* × *W*^2^)/2 (mm^3^).

### 4.20. Statistical Analysis

Data were expressed as the mean ± standard error (SE). Statistical analyses of the experimental data were performed using a two-sided Student’s *t*-test. *p* values < 0.05 were determined to indicate statistical significance.

## Data Availability

All original data and images are contained within the article.
